# TASP1 Promotes Gallbladder Cancer Cell Proliferation and Metastasis by Up-regulating FAM49B via PI3K/AKT Pathway

**DOI:** 10.7150/ijbs.40516

**Published:** 2020-01-14

**Authors:** Yijian Zhang, Pengcheng Du, Yang Li, Qin Zhu, Xiaoling Song, Shibo Liu, Jiaqi Hao, Liguo Liu, Fatao Liu, Yunping Hu, Lin Jiang, Qiang Ma, Wei Lu, Yingbin Liu

**Affiliations:** 1 Department of General Surgery, Xinhua Hospital Affiliated to Shanghai Jiao Tong University School of Medicine, 1665 Kongjiang Road, Shanghai 200092, China; 2 Shanghai Key Laboratory of Biliary Tract Disease Research, 1665 Kongjiang Road, Shanghai 200092, China; 3 Shanghai Research Center of Biliary Tract Disease, 1665 Kongjiang Road, Shanghai 200092, China; 4 Department of General Surgery, Second Affiliated Hospital of Nanchang University, 1 Minde Road, Nanchang 330006, China; 5 Department of Thyroid Oncology, Shanghai East Hospital Affiliated to Tongji University School of Medicine, 150 Jimo Road, Shanghai 200120, China.

**Keywords:** TASP1, Tumor progression, Gallbladder cancer, PI3K/AKT pathway, FAM49B

## Abstract

The highly conserved protease TASP1 not only takes part in critical site-specific proteolysis, but also plays an important role in numerous liquid and solid malignancies. However, the TASP1 expression and its biological regulation function in malignant gallbladder carcinoma (GBC) remain fully unknown. Here we observed that TASP1 levels were substantially overexpressed in GBC samples compared with non-tumor tissues. High TASP1 level was closely associated with T stage and metastasis, and was also correlated with poor prognosis in GBC patients. The depletion of TASP1 inhibited GBC cell proliferation and metastasis *in vitro* and *in vivo*. Furthermore, we first revealed that FAM49B had biological function and was positively regulated by TASP1 activating PI3K/AKT signaling pathway in GBC. At the same time, FAM49B also promoted GBC cell proliferation and migration. Inhibition of PI3K/AKT with LY294002 or FAM49B expression abrogated Myc-TASP1/Lv-shTASP1-induced GBC cell proliferation and motility. In conclusion, these findings demonstrate that TASP1 is critical for GBC progression via TASP1-PI3K/AKT-FAM49B axis and it may be a novel prognostic factor. The therapeutic targeting TASP1 may be a potential treatment approach for GBC patients.

## Introduction

Gallbladder cancer (GBC) is the most common biliary tract malignancy with an incidence of 2.5 per 1 × 10^5^ persons according to the Surveillance, Epidemiology, and End Results (SEER) program 1-5. Despite the relatively low incidence rate, owing to its nonspecific symptoms and highly invasive nature, GBC-associated mortality rate is higher than most other cancers 6-9. Although surgical resection is known as the most common and effective treatment, the 5-year survival rate of GBC patients is only 5% 10-14. Therefore, it is important to identify novel and effective potential biomarkers and therapeutic targets for the treatment of this disease, and increase our understanding of the pathogenesis and molecular mechanisms of GBC metastasis and recurrence for the development of effective adjuvant therapy.

Site-specific proteolysis is not only a critical requirement for life, but the executing enzymes also play an important role in numerous pathological conditions, including cancer 15-18. Therefore, targeting proteases is clearly relevant for exploring cancer therapy 19. The highly conserved protease Taspase 1 (threonine aspartase 1; TASP1) was initially identified as an endopeptidase responsible for cleaving mixed lineage leukemia 1 (MLL1) protein for proper regulation of HOX expression 20, 21. Other genetically and biochemically proven TASP1 substrates include MLL2, TFIIAα-β, ALFα-β, and *Drosophila* HCF-1 22-24. Interestingly, all of these substrates are broad-acting nuclear factors that regulate gene expression, suggesting a critical role for TASP1 in the orchestration of various biological processes 23. TASP1 drives cell cycle progression and coordinates cell proliferation and apoptosis 19. Aberrant expression of TASP1 has been found in numerous liquid and solid malignancies, including leukaemia, breast cancer, lung cancer, head and neck cancer, brain cancer, colon cancer, glioblastoma and melanoma 19, 25-28. Knockdown of TASP1 in many cancer cell lines impairs cancer cell proliferation and even sensitizes brain cancer and melanoma cells to anoikis 20. Although TASP1 has been found in various cancers and was characterized as a 'non-oncogene addiction' protease, our knowledge on its detailed functions and the underlying mechanisms contributing to cancer is still incomplete.

Family with sequence similarity 49 member B (FAM49B) is encoded by a highly conserved gene in mammals 29. This protein was previously thought to have no function. Recently, Chattaragada *et al*. found FAM49B acts as a suppressor of cancer cell proliferation and invasion in pancreatic ductal adenocarcinoma (PDAC) by regulating tumor mitochondrial redox reactions and metabolism, and Shang *et al*. found that FAM49B inhibits T cell activation by repressing Rac activity and modulating cytoskeleton reorganization 29, 30. These data indicate that FAM49B could play an important role in biological process and it may be associated with cancer progression. However, the pathological role of FAM49B in GBC remains unknown.

In this study, we first found that TASP1 displays significantly high expression and there exists a correlation between the high expression of TASP1 and poor prognosis in GBC patients. Second, TASP1 depletion effectively inhibits cell proliferation and metastasis *in vitro*, and markedly suppresses tumor growth *in vivo*. Last, we demonstrate for the first time that FAM49B has oncogenic biological function and is targeted and regulated by TASP1-PI3K/AKT pathway in GBC. This research identified the TASP1-PI3K/AKT-FAM49B axis, which is a new molecular pathway in GBC development, and may yield great clinical promise for GBC diagnosis and treatment.

## Materials and Methods

### Tissue specimens and clinicopathological data

This study was approved by the Xinhua Hospital Ethics Committee Affiliated to Shanghai Jiao Tong University School of Medicine (ID no: XHEC-D-2018-077 and XHEC-F-2018-025), and all patients provided informed consent. Tissue specimens were obtained from 72 GBC patients and 60 cholecystitis patients who underwent radical cholecystectomy without prior radiotherapy, chemotherapy or androgen therapy before surgery between 2011 and 2016 at the Department of General Surgery, Xinhua Hospital Affiliated to Shanghai Jiao Tong University School of Medicine, Shanghai, China. All diagnosis of GBC, cholelithiasis and lymph node metastasis were confirmed by hematoxylin and eosin (H&E) staining. Among the 72 gallbladder adenocarcinomas, the survival information for patients was collected through phone calls. In addition, 60 patients with cholelithiasis who underwent simple cholecystectomy were included as controls. Fresh GBC tissue samples and matched adjacent non-cancerous tissue samples were snap frozen and stored in liquid nitrogen for quantitative real-time PCR (qRT-PCR) assay. Each tissue sample was immediately fixed in 4% formalin after removal and embedded in paraffin for immunohistochemistry (IHC). All tissue samples were confirmed to be GBC or non-malignant tissues by two independent pathologists and staged according to the 7^th^ AGCC-TNM Classification of Malignant Tumors.

### IHC analysis of GBC tissues

IHC staining was performed following a standard procedure. Following deparaffinization and quenching of endogenous peroxidase, sections were incubated with 1% bovine serum albumin (BSA) in PBS. Subsequently, the sections were treated with anti-TASP1 and anti-FAM49B (1:250, Abcam, Burlingame, CA, USA) antibodies followed by incubation with a goat anti-rabbit-peroxidase-conjugated second antibody (Santa Cruz). DAB (3,3-diaminodbenzidine) substrate (Dako, Carpinteria, CA, USA) was added and the sections were counterstained with hematoxylin. Finally, the sections were mounted and observed under a microscope (Leica, Wetzlar, Germany).

Sections were semi-quantitatively scored as described previously 31. The percentage of positive staining cells were scored as follows: 0 for no staining, 1 for <5% immunoreactive cells; 2 for 5-50% immunoreactive cells; and 3 for >50% immunoreactive cells. In addition, the staining intensity was graded as 0 for no staining, 1 for weak immunoreactivity, 2 for intermediate immunoreactivity and 3 for strong immunoreactivity. The samples were grouped based on the sum of both extension and intensity parameters: negative (0), weak (1-2), moderate (3), and strong (4-6) staining.

### Cell culture and reagents

The human GBC-SD cell lines and the normal biliary epithelia cell line HIBEC were purchased from the Cell Bank of the Chinese Academy of Sciences (Shanghai, China), and EH-GB-1 and NOZ cell lines were purchased from the Health Science Research Resources Bank (Osaka, Japan). The another human GBC cell line, SGC-996, was obtained from the Tongji University School of Medicine (Shanghai, China). The GBC-SD and EH-GB-1 cells were cultured in high-glucose DMEM (Gibco, Grand Island, NY, USA), NOZ cell line was maintained in William's medium (Gibco, Grand Island, NY, USA), and SGC-996 cell line was grown in RPMI 1640 (Hyclone, Logan, TX, USA) in a humidified incubator (5% CO_2_, 37^ o^C). Cells were cultured in recommended medium supplemented with 10% (v/v) fetal bovine serum (Gibco, USA), 100 μg/mL streptomycin and 100 U/mL penicillin (Hyclone).

LY294002 (S1105) was purchased from Selleck Chemicals (Houston, TX, USA), and GBC-SD and EH-GB-1 cell lines were treated with LY294002 at 20 μM for 12 h.

### RNA interference and plasmid construction

pGMLV-SC5 lentivirus core vectors contain a GFP reporter and puromycin resistance, and encode short hairpin RNA (shRNA) targeting negative control (5'-TTCTCCGAACGTGTCACGT-3'), TASP1 (5'-CAGAUUUUAUGCAACUAAA-3') and FAM49B (5'-GAGACTACCTCCAAGCAAA-3'), respectively, which were provided by Genechem (Shanghai, China). Cells were transfected with lentivirus at an MOI (multiplicity of infection) of 40 for 48 h and then selected with puromycin (1 μg/mL) for 3 days.

The full-length sequences of TASP1 (NM_017714) and FAM49B (NM_001256763) cDNA were amplified and subcloned separately into the pCMVPuro02 and GV141 expression vectors using the primers listed in Table S1. These plasmids were transfected into GBC cells using ViaFect™ transfection reagent following the manufacture's instructions (Promega), and the expression levels in transfected cells were compared with empty vector transfected cells (MOCK).

### Quantitative real-time PCR

Total RNA from tissues and cells was extracted with Trizol reagent (Invitrogen, Carlsbad, CA, USA). The cDNA was synthesized using a reverse transcription reagent kit (TaKaRa, Dalian, China). Target genes were amplified by qRT-PCR using the SYBR-Green method (TaKaRa, Dalian, China) according to the manufacturer's instructions. The products were detected by StepOnePlus™ Real-Time PCR system (Applied Biosystems). GAPDH was served as an internal control. Each sample was tested from three independent experiments. Table S2 shows the primer sequences for qRT-PCR.

### Western blots

Proteins from cell lysates were separated by SDS-PAGE and then transferred onto 0.45 μm PVDF membranes (Millipore). The PVDF membranes were blocked with 5% skim milk and then incubated with a series of primary antibodies. The anti-TASP1 (ab63160), -FAM49B (ab121299) and -p-PI3K (Tyr607, ab182651) antibodies were acquired from Abcam (Cambridge, MA, USA). Anti-E-cadherin (#3195), -N-cadherin (#13116), -vimentin (#5741), -PI3K (#4249), -AKT (#9272), -p-AKT (Ser473, #4060) and -GAPDH (#2118) antibodies were purchased from Cell Signaling Technology (Danvers, MA, USA). After washing three times with TBST buffer, the membranes were incubated with appropriate HRP-conjugated secondary antibodies and the bands were visualized using an enhanced chemiluminescent detection reagent from Pierce (Rockford, IL, USA). All experiments were independently repeated three times.

### Cell proliferation and colony formation assays

Cell proliferation was examined by a Cell Counting Kit-8 (CCK-8) (Yeasen, Shanghai, China) cell proliferation assay following the manufacturer's instructions. The transfected cells in 100 μL medium were seeded into 96-well plates (1,000 cells in each well). At each indicated time point (1 d, 2 d, 3 d, 4 d, 5 d), the number of viable cells was assessed by adding 10 μL CCK8 (Dojindo, Kumamoto, Japan) to each well, and the cells were incubated for 3 h at 37 °C. Absorbance was measured at 450 nm using a microplate reader (Bio-TEK, Saxony, USA) to obtain growth curve of GBC cells. To perform colony formation assay, GBC cells were seeded in six-well plates (400 cells/well). After culturing for 2 weeks, colonies were fixed with 4% paraformaldehyde for 30 min and stained with 0.1% crystal violet (Beyotime) for 20 min. Then stained colonies were photographed and the numbers of colonies (>50 cells/colony) were counted after rinsing three times with PBS. Three independent experiments were performed.

### Transwell cell migration and invasion assays

Cell motility was examined using 8.0-μm pore transwell filters (Corning, NY, USA), and invasion assay was evaluated using matrigel ® invasion chamber (BD Biosciences, Franklin Lakes, NJ, USA) in the 24-well plate. 2 × 10^4^ cells in serum-free medium were plated into the upper chamber of the transwell plate or the invasion chamber, while the lower chamber was filled with 500 μl of medium supplemented with 10% FBS. After 20 h of incubation, cells that migrated to the bottom of the insert were fixed with 4% paraformaldehyde and stained with 0.1% crystal violet. We chose five random fields to capture images and counted cells at 100× magnification under a microscope. Transwell cell migration and invasion assays were independently performed in triplicate.

### mRNA microarray analysis

The total RNA of GBC-SD cells transfected with Lv-shNC/Lv-shTASP1 was extracted, amplified and labelled by Low Input Quick Amp Labeling Kit, One-Color (Agilent technologies, Santa Clara, CA, USA) according to the manufacturer's instructions. After hybridization and staining, slides were scanned by Agilent Microarray Scanner (Agilent technologies, Santa Clara, CA, USA). Data were normalized and statistical analyzed using the paired t-test. The threshold we set to screen for upregulated and downregulated genes was a fold change ≥ 1.2 and a *P*-value ≤ 0.001. Hierarchical clustering was performed and a heat map was constructed with TreeView (http://jtreeview.sourceforge.net) to visualize differentially expressed mRNA.

### Nude mouse model of subcutaneous xenograft

All procedures including the purchase of nude mice and collection of samples were approved by the Xinhua Hospital Ethics Committee Affiliated to Shanghai Jiao Tong University School of Medicine (ID no: XHEC-F-2018-025). 1×10^6^ GBC-SD cells in 100 μl PBS (Lv-shNC/Lv-shTASP1 group) were subcutaneously injected into the left axilla of 4-6 weeks old male nude mice (five mice/group) under anesthesia. Tumor length and width were measured every week following tumor development. Tumor volume was calculated according to the equation: tumor volume (mm^3^) = 1/2 × width^2^ (mm^2^) × length (mm). The corresponding tumors were weighed when the mice were sacrificed, and then harvested tissues were used for IHC staining analysis.

### Statistical analysis

SPSS software (version 19.0) and GraphPad Prism software (version 6.0) were used for statistical analyses. TASP1 and FAM49B mRNA levels in tumor and paired non-tumor tissues were compared using paired Student's t-tests. The independent Student's t-test was used to compare the means of two groups. The associations between TASP1 expression and clinicopathologic parameters were determined using Chi-square test. Kaplan-Meier plots and log-rank tests were used for the survival analysis. The data (mean ± SD) were representative of at least three independent experiments, and *P* < 0.05 were considered statistically significant.

## Results

### TASP1 is overexpressed in GBC tissues and correlated with poor prognosis in GBC patients

**Figure 1:**
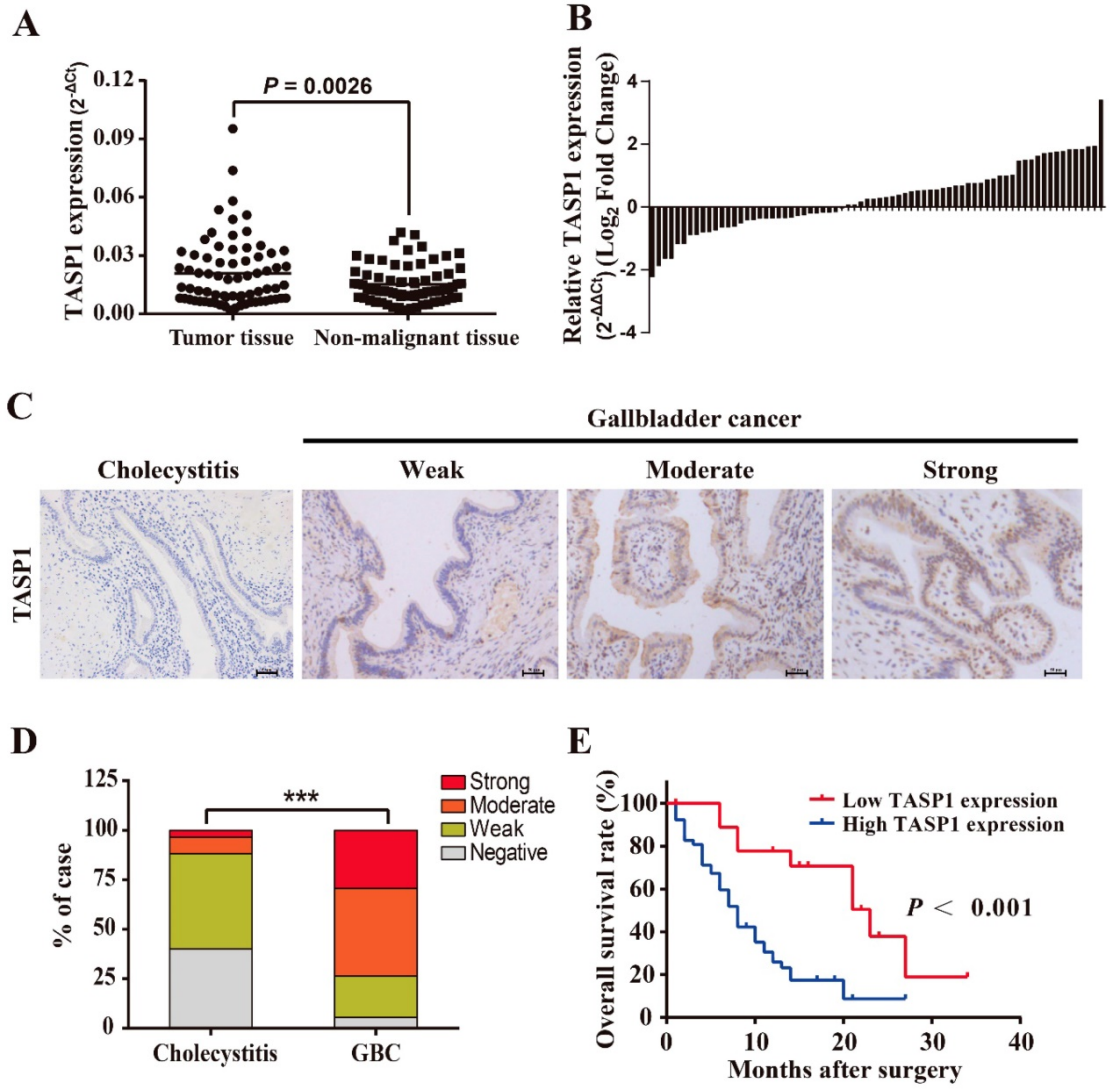
** TASP1 is overexpressed in GBCs and correlated with poor survival of GBC patients. (A)** TASP1 expression in GBC tissues and matched non-malignant tissues was evaluated by qRT-PCR. **(B)** TASP1 expression levels were compared between GBC tissues and their corresponding adjacent tissues. **(C)** IHC analysis of TASP1 protein expression level (scale bar, 50 μm). Representatives images of cholecystitis and GBC with weak, moderate, strong staining. **(D)** The percentage of different TASP1 staining in the cholecystitis and GBC tissues. **(E)** Kaplan-Meier overall survival curve of GBC patients based on TASP1 expression. Low TASP1, n=19; high TASP1, n=53.

To explore the pathological role of TASP1 in GBC development, we examined the TASP1 mRNA levels in 72 pairs of GBCs and found that the TASP1 expression was higher in tumor tissues compared with their corresponding adjacent non-malignant tissues (*P* = 0.0026) (Figure 1A and B). Furthermore, we assessed TASP1 expression levels in the gallbladder tumor and non-tumor tissues by IHC staining. The protein expression level of TASP1 was significantly increased in 72 GBC tissues as compared with the 60 cholecystitis tissues (Figure 1C). 29.2% (21/72) of the GBC samples exhibited strong staining, 44.4% (32/72) moderate staining, 20.8% (15/72) weak staining, and 5.6% (4/72) negative staining in the tumor samples; only 3.3% (2/60) of the cholecystitis specimens showed strong staining, 8.3% (5/60) moderate staining, 48.3% (29/60) weak staining, and 40.0% (24/60) negative staining of TASP1 protein, indicating that the TASP1 expression level was higher in tumor tissues (*P* < 0.001) (Figure 1D). The GBC patients were classified into TASP1-high (score ≥ 3) and TASP1-low (score < 3) groups according to a semi-quantitative assessment. We analyzed the association between TASP1 expression levels and clinicopathological characteristics from GBC patients and found that TASP1 expression level was significantly correlated with T stage (*P* = 0.004) and metastasis (*P* < 0.001) (Table 1). Moreover, the Kaplan-Meier analysis indicated that patients in TASP1-low group was significantly better than patients in TASP1-high (*P* < 0.001) (Figure 1E). These results suggest that upregulation of TASP1 is significantly associated with the progression of GBC pathogenesis.

### TASP1 promotes GBC cell proliferation *in vitro* and *in vivo*

**Figure 2:**
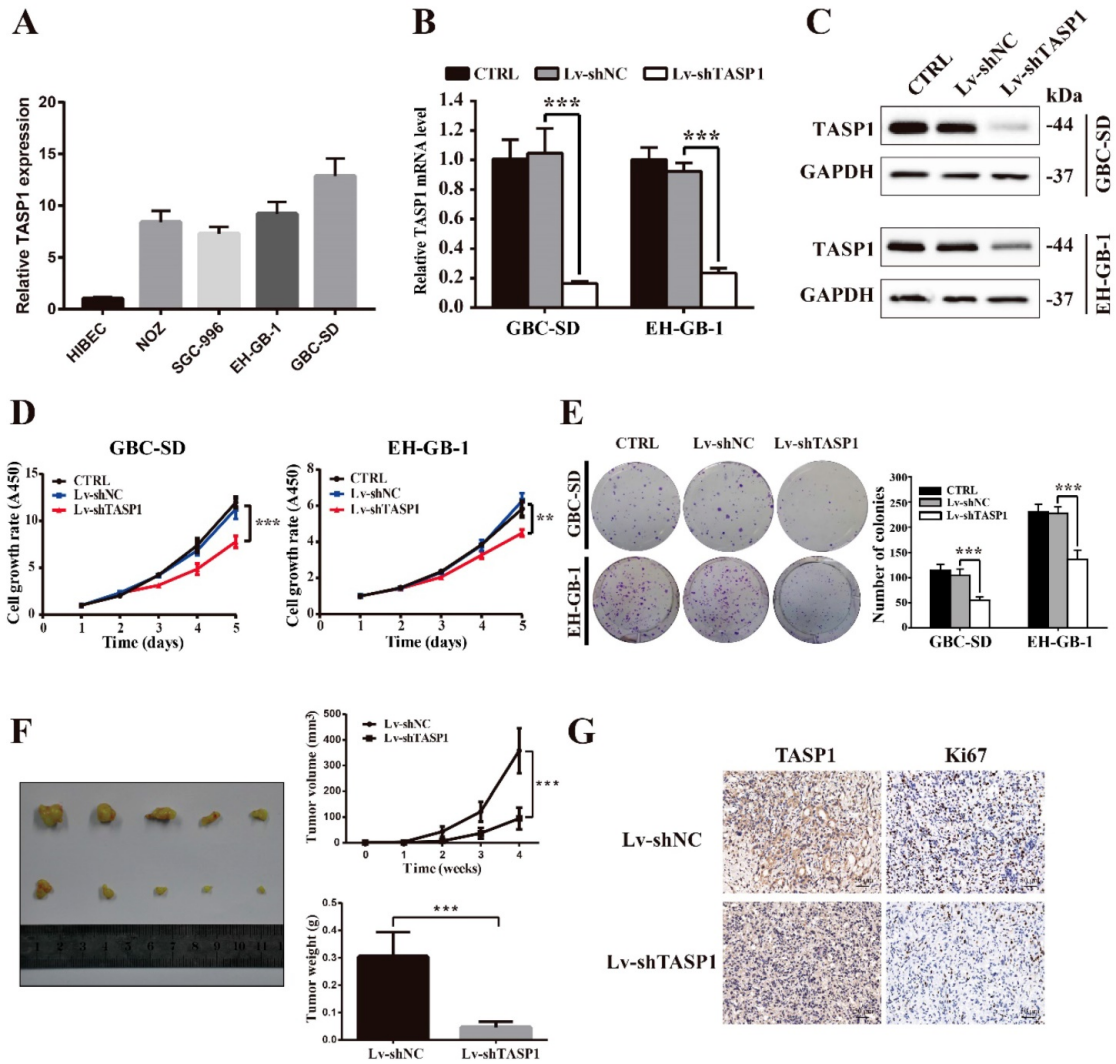
** TASP1 promotes GBC cell proliferation and tumor growth. (A)** mRNA expression of TASP1 in the HIBEC and GBC cell lines including NOZ, SGC-996, EH-GB-1 and GBC-SD. **(B-C)** mRNA and protein expression levels of TASP1 were examined in GBC-SD and EH-GB-1 cells transfected with Lv-shTASP1. **(D)** The proliferation of treated GBC cells was measured by CCK-8 assay. **(E)** The colony formation was assessed in GBC cells and statistical significance was analyzed based on the numbers of colonies. **(F)** The tumor size and weight were measured, which were formed in nude mice injected with the Lv-shNC- and Lv-shTASP1-GBC-SD cells.** (G)** The expression levels of TASP1 and Ki67 were detected in GBC subcutaneous xenograft model by IHC (scale bar, 50 μm). All results (mean ± SD) are from three separate experiments; **P < 0.01, ***P < 0.001.

To explore the biological roles of TASP1 in GBC development, we detected the endogenous expression of TASP1 in four GBC cell lines and a normal biliary epithelia cell line (Figure 2A). Consistent with the tumor tissue profiles, the GBC cells expressed higher levels of TASP1 than that in normal cell. Because TASP1 overexpression is correlated with poor prognosis in GBC patients, we chose GBC-SD and EH-GB-1 cell lines which have relatively high TASP1 expression level to explore oncogenic function (Figure 2A). The GBC-SD and EH-GB-1 cells were transfected with Lv-shTASP1 and Lv-shNC, and the expression of TASP1 was dramatically inhibited at both mRNA and protein levels (Figure 2B and C).

To investigate whether TASP1 affects GBC cell proliferation, we performed CCK-8 and colony formation assays. As shown in Figure 2D, the proliferation ability of GBC-SD and EH-GB-1 cells transfected with Lv-shTASP1 was significantly suppressed compared with control cells. In addition, TASP1 knockdown attenuated the colony formation capability of GBC cells (Figure 2E).

To determine the effect of TASP1 on gallbladder tumor progression *in vivo*, we performed xenograft tumor assays using TASP1-depleted GBC-SD cells. We found that the tumor volume and weight of TASP1-depleted xenografts were significantly inhibited (Figure 2F). IHC staining indicated that the expression level of the proliferative index Ki67 was higher in the Lv-shNC group than the Lv-shTASP1 group (Figure 2G). Collectively, these data indicate that TASP1 promotes GBC cell proliferation *in vitro* and *in vivo*.

### TASP1 promotes GBC cell migration and invasion *in vitro*

**Figure 3:**
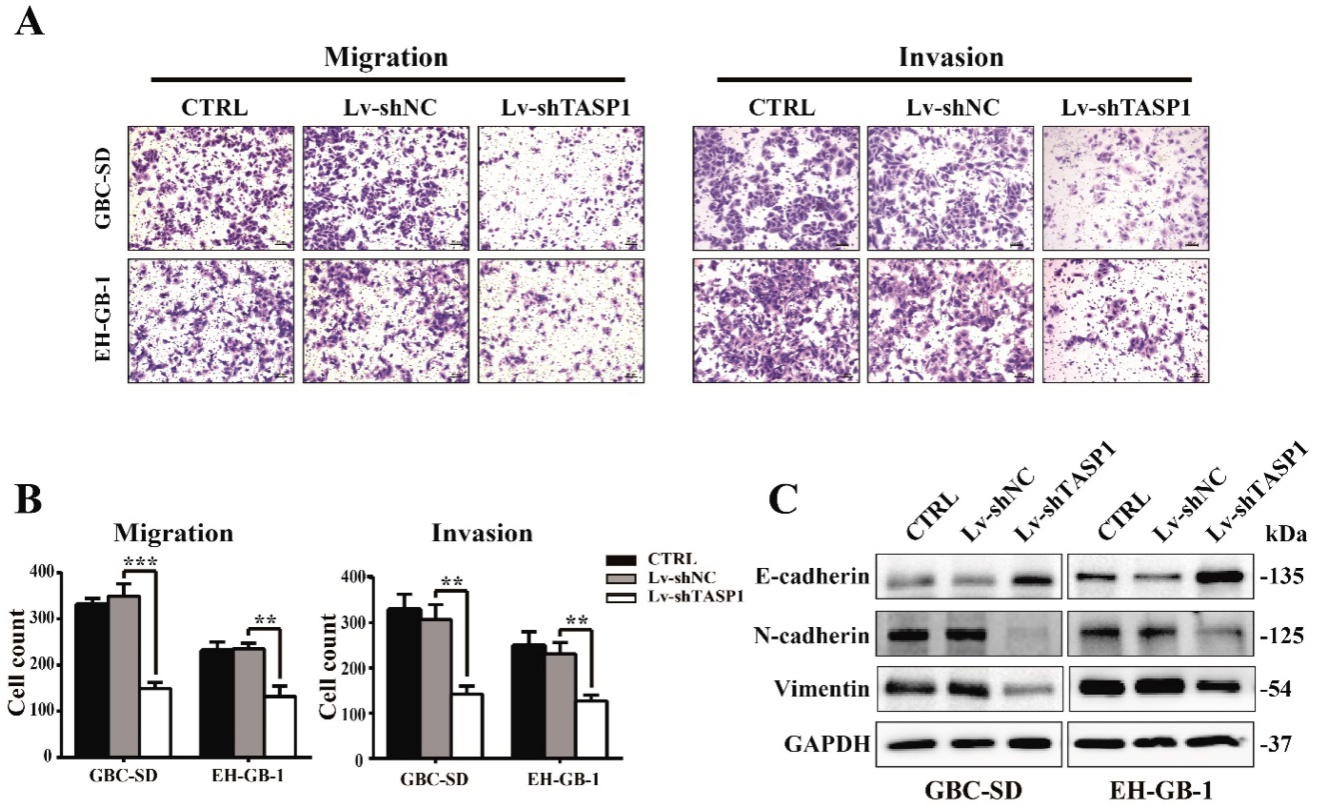
** TASP1 promotes GBC cell metastasis by inducing EMT. (A-B)** The migration (left panel) and invasion (right panel) conditions of treated GBC-SD and EH-GB1 cells were measured (scale bar, 100 μm). Statistical significance was analyzed according to the number of invaded cells; **P < 0.01, ***P < 0.001. **(C)** The protein expression levels of E-cadherin, N-cadherin and vimentin in the GBC-SD and EH-GB-1cells were examined by western blot.

The clinicopathologic association study in GBCs revealed that overexpression of TASP1 was significantly associated with metastasis, which implies that TASP1 may have a role in metastasis (Table 1). To explore if and how ectopic TASP1 overexpression promotes GBC metastasis, we conducted cell migration and invasion assays *in vitro*. As shown in Figure 3A and B, the migrative and invasive capability of cells transfected with Lv-shTASP1 was dramatically attenuated compared with control group, suggesting that TASP1 enhances metastatic capacity of GBC. Many researches have focused on the epithelial origin of cancer in terms of epithelial-mesenchymal transition (EMT). We examined related EMT biomarker expression using western blots and found that knockdown of TASP1 enhanced the protein level of E-cadherin (which is the characteristic marker of epithelial cells), whereas reduced N-cadherin and Vimentin levels (indicating a mesenchymal phenotype) (Figure 3C). Therefore, these data suggest that TASP1 affects GBC cell migration and invasion through EMT process.

### FAM49B is overexpressed in GBC tissues and has positively correlation with TASP1 expression in GBC patients

**Figure 4:**
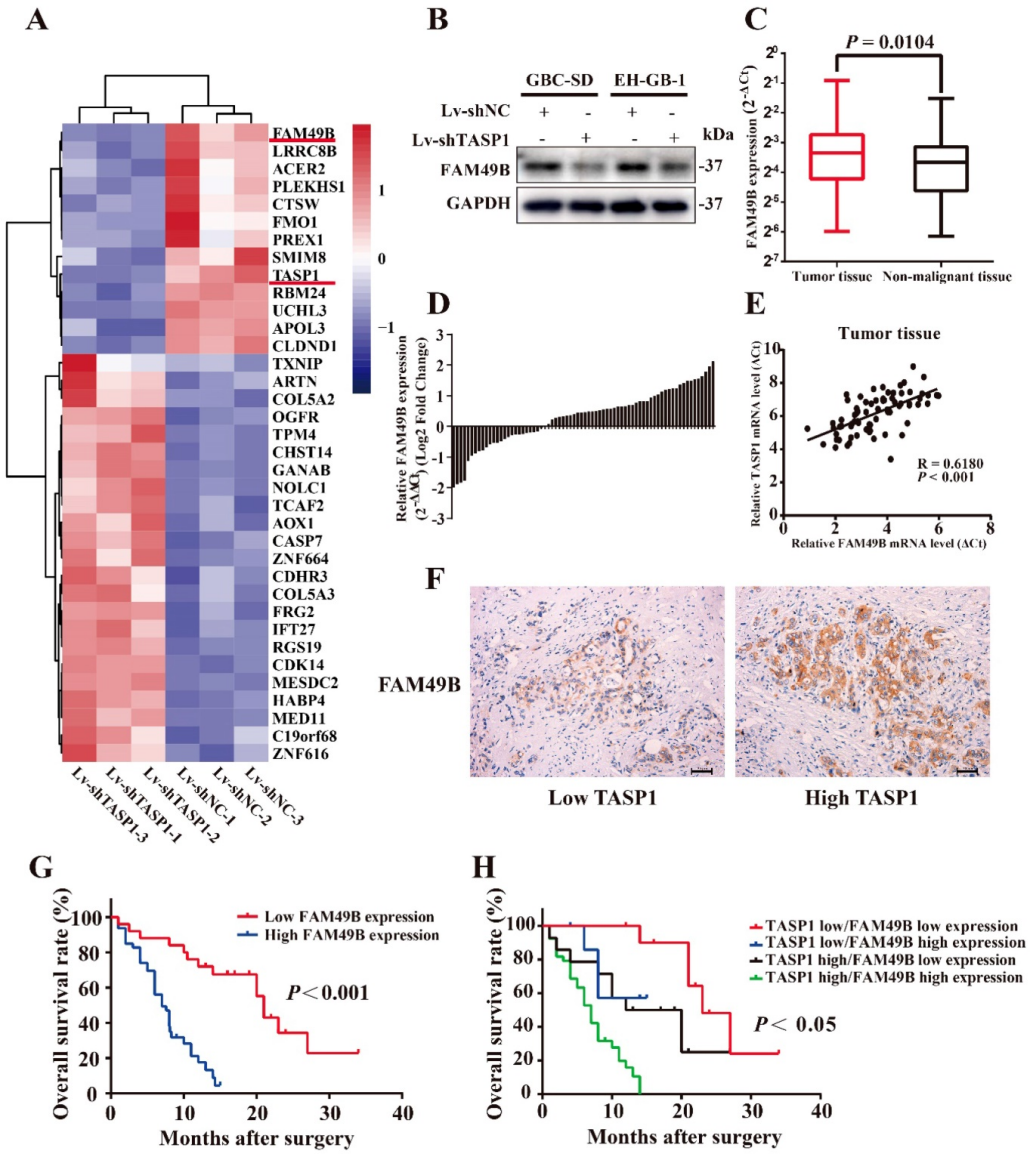
** FAM49B is overexpressed and has a positive correlation with TASP1 expression in GBC patients. (A)** A portion of cluster analysis of mRNAs that were differential expressed between Lv-shNC- and Lv-shTASP1-transfected GBC-SD cells. Red and blue represent high- and low-expression, respectively. **(B)** FAM49B expression was downregulated in Lv-shTASP1 group compared with Lv-shNC group. **(C)** FAM49B expression in GBC tissues and matched non-malignant tissues was evaluated by qRT-PCR. **(D)** FAM49B expression levels were compared between GBC tissues and their corresponding adjacent tissues. **(E-F)** The mRNA and protein expression levels of FAM49B were positively correlated with TASP1 expression levels. **(G)** Kaplan-Meier overall survival curve of patients based on high and low FAM49B expression. **(H)** The overall survival rates of 72 GBC patients were compared between the high/low TASP1 expression groups and the high/low FAM49B expression groups by Kaplan-Meier analysis.

To explore the molecular mechanism by which TASP1 promotes the proliferation and metastasis of GBC cells, we performed a mRNA microarray assay to compare the mRNA expression profiles of Lv-shNC and Lv-shTASP1 groups. The results showed significant expression alterations (Figure 4A). Among these candidates, we found that the expression level of FAM49B, which was previously thought to have no function, was dramatically downregulated in Lv-shTASP1 group compared with Lv-shNC group. To further validate the functional interaction between TASP1 and FAM49B in the cells, we examined whether ectopic expression of TASP1 could alter the expression of FAM49B. We found that FAM49B protein expression level was decreased in Lv-shTASP1 group compared with Lv-shNC group (Figure 4B). However, TASP1 expression level was not affected in the FAM49B knockdowned cells (Figure S1). These data indicated that TASP1 had positive regulatory effect on FAM49B expression.

To further explore the clinical relevance of FAM49B in GBC, we firstly detected the mRNA levels of FAM49B in 72 pairs of GBCs by the qRT-PCR assay and found that FAM49B expression was significantly increased in GBC tissues compared with their corresponding adjacent non-malignant tissues (Figure 4C and D). The TASP1 and FAM49B mRNA levels has a significant positively correlation in tumor specimens by Pearson correlation analysis (R = 0.6180, *P* < 0.001; Figure 4E). Secondly, we tested FAM49B protein expression level using IHC staining, and found that GBC patient tumors with high TASP1 levels showed strong FAM49B staining, whereas tumors with low TASP1 levels showed weak FAM49B staining (Figure 4F, Table 2). Furthermore, elevated expression of FAM49B among the 72 GBC specimens also predicted poor patient prognosis (Figure 4G). Those patients with both elevated levels of TASP1 and FAM49B exhibited more worse prognosis (Figure 4H). Collectively, these results indicated that TASP1/FAM49B has an important role in the progression of GBC and may be a pair of novel prognostic factors for predicting the clinical recurrence and poor survival of GBC patients.

### TASP1 promotes GBC cell proliferation and migration by upregulating FAM49B

**Figure 5:**
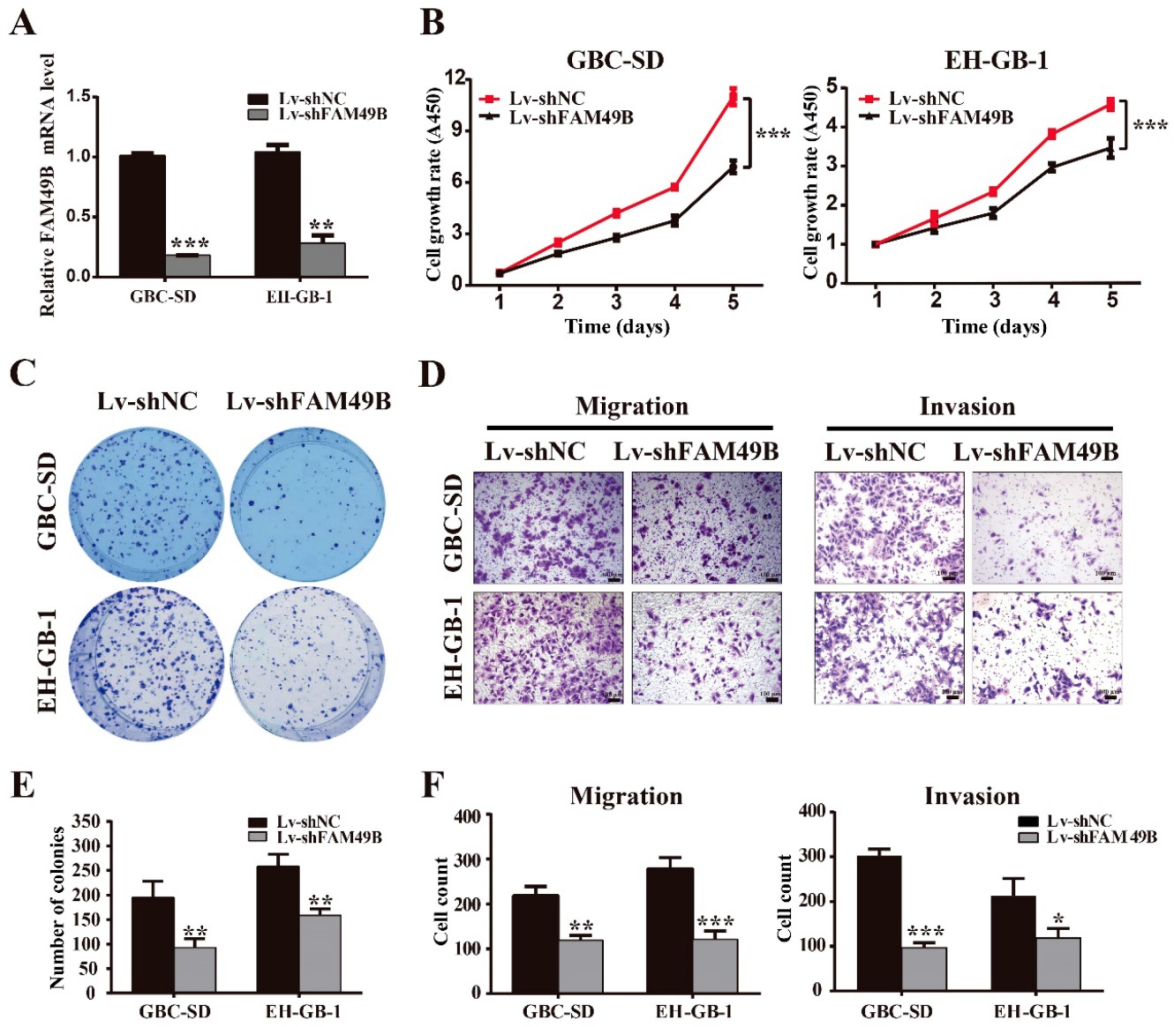
** FAM49B promotes GBC cell proliferation and metastasis. (A)** The mRNA expression level of FAM49B was downregulated in GBC-SD and EH-GB-1 cells transfected with Lv-shFAM49B. **(B-C)** Cell proliferation and colony formation abilities were inhibited in GBC-SD and EH-GB-1 cells transfected with Lv-shFAM49B.** (D)** Knockdown of FAM49B suppressed the migration and invasion abilities of the GBC-SD and EH-GB-1 cells. **(E-F)** Statistical significance of colony formation and metastasis assays was analyzed according to the number of colony and invaded cells; **P < 0.01, ***P < 0.001.

FAM49B was previously thought to have no function in many tissues. To determine whether the TASP1-dependent promotion of GBC cell proliferation and metastasis was indeed mediated by FAM49B, we first examined whether FAM49B had some biological functions in GBC cells. We first transfected GBC cells with Lv-shNC and Lv-shFAM49B, respectively (Figure 5A). The data indicated that the cell proliferation and colony formation were reduced in the GBC-SD and EH-GB-1 cells transfected with Lv-shFAM49B compared to negative controls, and cell migration and invasion abilities were also significantly decreased in Lv-shFAM49B group compared to Lv-shNC group (Figure 5B-F). Collectively, these results demonstrate that FAM49B plays a protumorigenic role in GBC cells.

**Figure 6:**
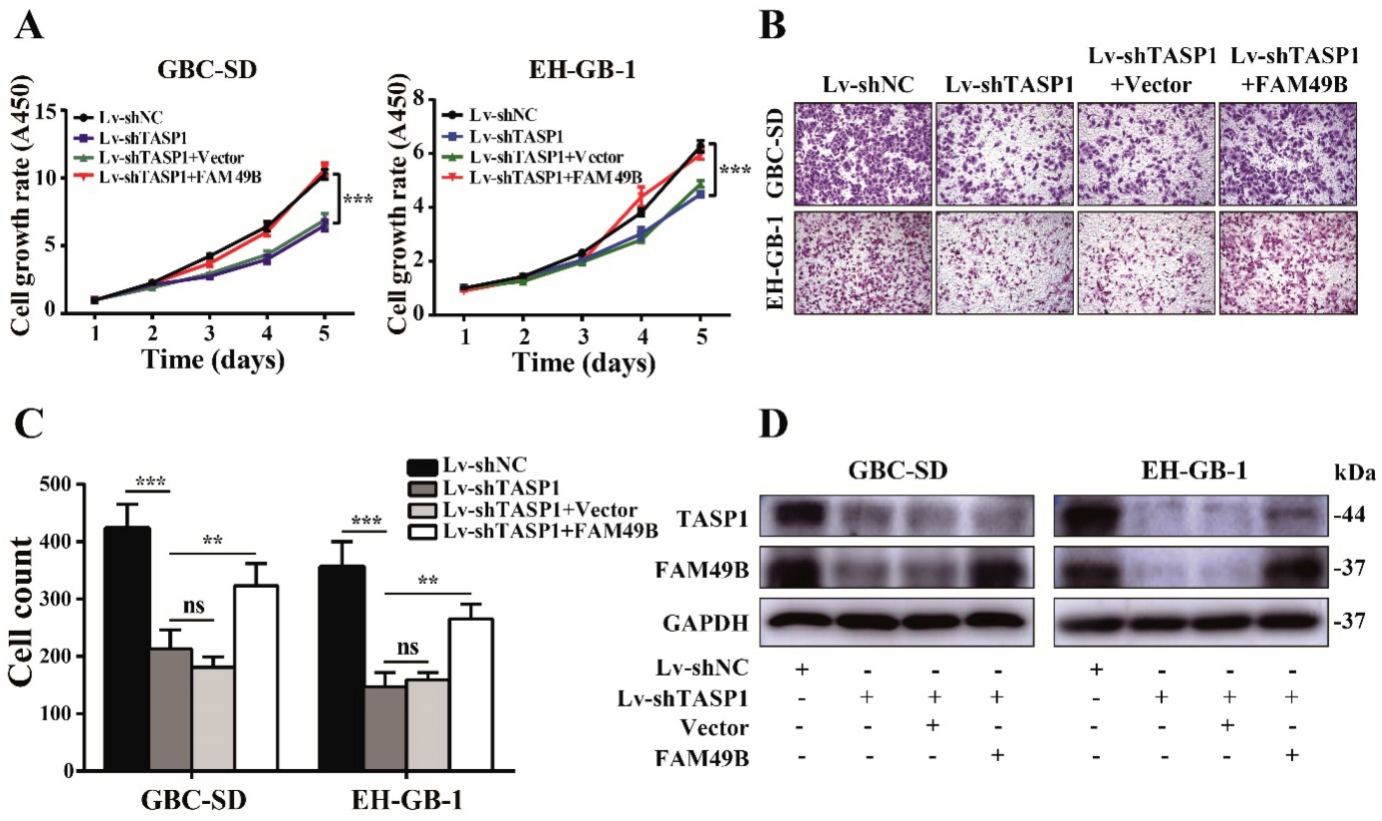
** FAM49B attenuates the effect of the knockdown of TASP1 on GBC. (A-C)** The grow rate and metastasis ability were examined in the Lv-shTASP1 group transfected with/without FAM49B plasmid. **(D)** The TASP1 and FAM49B expression levels were determined in Lv-shNC and Lv-shTASP1 groups transfected with vector or FAM49B plasmid by western blot. GAPDH was used as the loading control.

We next used FAM49B vector to illustrate the function of FAM49B in TASP1-mediated GBC progression. First, the GBC-SD cell was transfected with Lv-shNC/Lv-shTASP1, and as expected, cell growth rate and motility were reduced when TASP1 expression was depleted. We then transfected Lv-shNC/Lv-shTASP1 groups with the FAM49B vector and found a promoting effect on growth and migration of GBC-SD cells (Figure 6A-D). Thus, these results indicate that FAM49B represents an important target in the underlying mechanism of the TASP1-mediated GBC development.

### TASP1 regulates FAM49B by activating the PI3K/AKT signaling pathway

**Figure 7:**
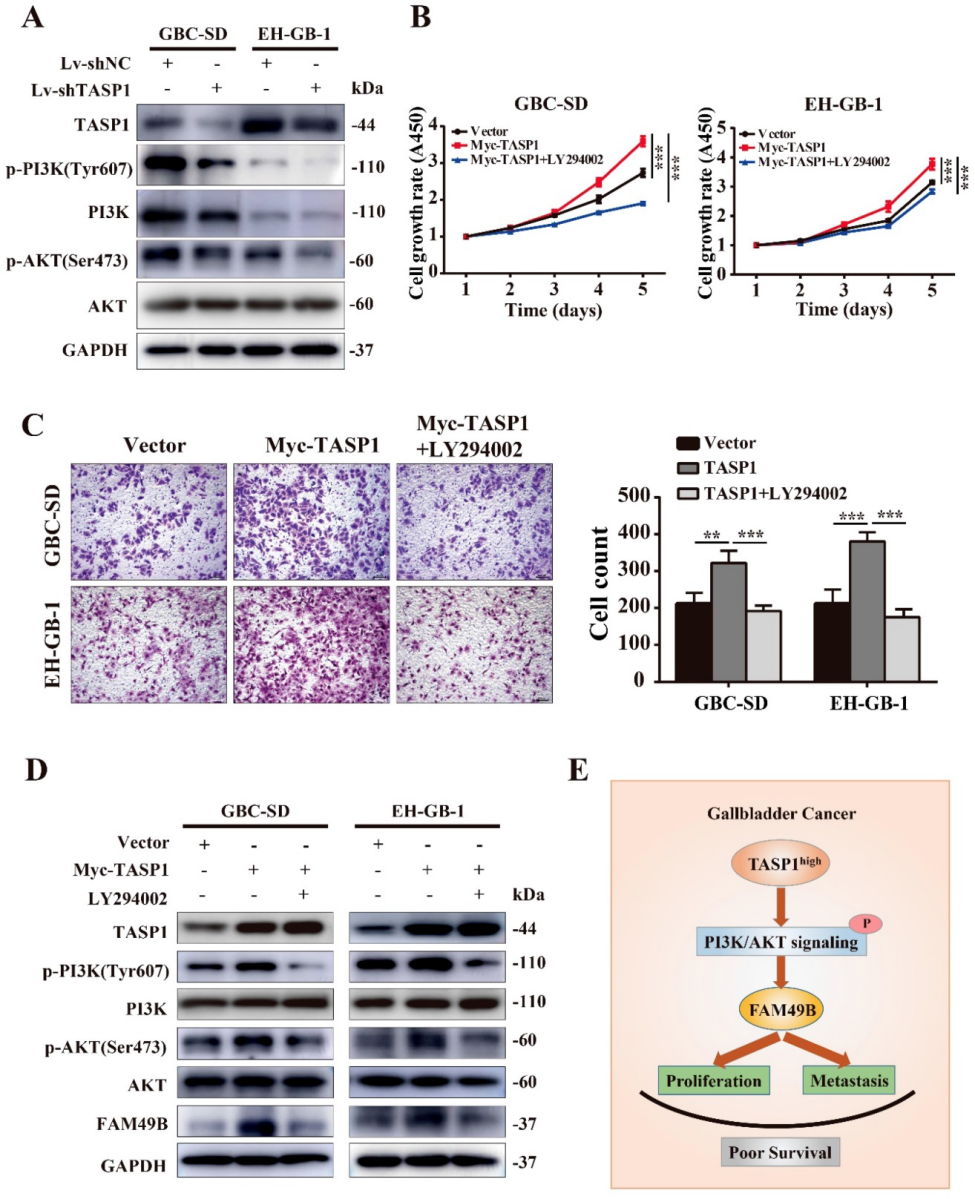
**TASP1 regulates FAM49B via the PI3K/AKT signaling pathway. (A)** The expression levels of TASP1, phosphorylated PI3K (Tyr607)/total PI3K and phosphorylated AKT (Ser473)/total AKT were determined in Lv-shNC and Lv-shTASP1 groups by western blot. **(B-D)** Myc-TASP1-transfected GBC cells was treated with 20 μM LY294002 for 12 h. The proliferation and migration abilities were reduced. TASP1, phosphorylated PI3K (Tyr607)/total PI3K, phosphorylated AKT (Ser473)/total AKT and FAM49B expression levels were examined by western blot. GAPDH was used as the loading control. **(E)** The proposed mechanistic scheme of TASP1 regulating FAM49B by activating PI3K/AKT signaling pathway in GBC.

It is worth mentioning that the MLL exerts the regulation role after proteolytic cleavage by TASP1 in many cancers, and PI3K/AKT signaling pathway is involved in the MLL mediated process 32, 33. To investigate the molecular mechanism of TASP1-mediated upregulation of FAM49B, we first examined the PI3K/AKT signaling pathway. We found that p-PI3K (Tyr607) and p-AKT (Ser473) levels were markedly decreased in the GBC-SD and EH-GB-1 when TASP1 was depleted (Figure 7A). It suggests that the PI3K/AKT signaling pathway may participate in TASP1-mediated GBC progression.

To further support the role of PI3K/AKT pathway in the TASP1 regulating FAM49B activity, we tested the difference in the TASP1-overexpressing GBC-SD and EH-GB-1 cells treated with and without PI3K/AKT inhibitor (LY294002). The proliferation and migration abilities of GBC cells were downregulated in treated group (Figure 7B and C). p-PI3K (Tyr607), p-AKT (Ser473) and FAM49B levels were downregulated, but TASP1 level was significantly unchanged after treatment with LY294002 (Figure 7D). These data show that the TASP1 regulates FAM49B by activating the PI3K/AKT signaling pathway.

## Discussion

TASP1 is classified as a “non-oncogene addiction” protease and plays an important role in various cancer development 20, 23. Herein, we for the first time found that TASP1 is highly expressed in GBC tissues compared with the matched non-malignant tissues. TASP1 overexpression in GBC patients is correlated with T stage, metastasis and a shorter OS time based on clinicopathological data. In addition, our results also demonstrated that TASP1 remarkably promotes the proliferation and metastasis capacity of GBC cells *in vitro* and *in vivo*, which indicates that TASP1 is involved in GBC development and may be an important biomarker and therapeutic target in GBC patients.

TASP1 was first identified in 2003 as the protease responsible for the cleavage of the MLL protein to regulate HOX expression 19, 21. Many studies determined TASP1 is characterized as a nuclear protease accumulating at the nucleolus 19, 34. However, we found that TASP1 is located in the nucleus and cytoplasm in GBC tissues (Figure 1C). It indicates that there may exist the underlying regulatory mechanism in GBC different from other cancer. To define the regulatory scenario, we performed a mRNA microarray assay and identified for the first time that TASP1 positively regulates FAM49B function.

The FAM49B protein was previously characterized with no function and role in cancer 35. This protein is only recently determined as a suppressor that links the inflammatory environment to mitochondrial dynamics in PDAC 29. However, we found that FAM49B expression is increased in GBC tissues compared with the matched non-malignant tissues and there exists a positive correlation between TASP1 and FAM49B expression level. Knockdown of FAM49B decreases the proliferation and migration capacity of GBC cells. The growth and metastasis tendency of GBC-SD and EH-GB-1 cells transfected with Lv-shTASP1 could be attenuated by upregulating FAM49B expression. These data show that FAM49B acts as a oncoprotein of GBC cell proliferation and metastasis and is regulated by upstream TASP1.

Activated PI3K/AKT modulates the function of numerous substrates involved in the regulation of cell survival, cell cycle progression and cellular growth, and PI3K/AKT signaling pathway is critical in human cancer development 36, 37. Although PI3K signaling increases MLL1 cleavage by TASP1 and MLL1 activity in prostate cancer, we determined that TASP1 regulated FAM49B expression through PI3K/AKT signaling pathway in GBC cell lines. Whether this signaling pathway is located at the downstream of TASP1 may be based on the cancer type and tissue specificity 33. We treated GBC-SD and EH-GB-1 cells overexpressing TASP1 with LY294002 and found the proliferation and metastasis abilities of GBC cells, and FAM49B expression were attenuated. This supports our conclusion that TASP1 positively upregulates FAM49B expression by PI3K/AKT signaling pathway to mediate GBC cancer cell proliferation and migration.

In conclusion, our study for the first time suggests the clinical and biological significance of TASP1 and FAM49B in GBC progression. We also provide further evidence that TASP1 is critical for GBC progression via TASP1-PI3K/AKT-FAM49B axis (Figure 7E). TASP1 may be used as a prognostic biomarker and pharmacological targeting TASP1 may provide a potential treatment approach for GBC patients.

## Supplementary Material

Supplementary figure and tables.

## Figures and Tables

**Table 1:** Comparison of clinicopathological profiles of GBC patients between the low and high TASP1 expression groups

Variables	TASP1 expression level		Total (n=72)	*P* value
	Low (n=19)	High (n=53)		
*Age*				0.181
<60	3	19	22	
≥60	16	34	50	
*Gender*				0.326
Male	10	21	31	
Female	9	32	41	
*Differentiation*				0.124
High	6	6	12	
Moderate	9	31	40	
Low	4	16	20	
*T stage*				0.004*
Tis-T2	9	8	17	
T3-T4	10	45	55	
*Metastasis*				<0.001*
N0	17	20	37	
N1/N2	2	33	35	

Statistical analyses were performed with the Chi-square test. *P <0.05 was considered statistically significant.

**Table 2:** Correlation between TASP1 and FAM49B expression levels in GBC patients

TASP1 staining intensity	FAM49B staining intensity		χ2 value	P value
	Low (n=25)	High (n=47)		
Low (n=19)	11	8	6.115 0.013	
High (n=53)	14	39		

Statistical analyses were performed with the Chi-square test. *P <0.05 was considered statistically significant.

## Abbreviations

GBC: gallbladder carcinoma
SEER: Surveillance, Epidemiology, and End Results
TASP1: threonine aspartase 1/taspase 1
MLL1: mixed lineage leukemia 1
FAM49B: family with sequence similarity 49 member B
PDAC: pancreatic ductal adenocarcinoma
H&E: hematoxylin and eosin
qRT-PCR: quantitative real-time PCR
IHC: immunohistochemistry
BSA: bovine serum albumin
shRNA: short hairpin RNA
MOCK: empty vector transfected cells
CCK-8: Cell Counting Kit-8
