# Supplementary material for: TASP1 Promotes Gallbladder Cancer Cell Proliferation and Metastasis by Up-regulating FAM49B via PI3K/AKT Pathway
Source: Int J Biol Sci. 2020 Jan 14;16(5):739–51. doi: 10.7150/ijbs.40516 (PMC7019140; doi:10.7150/ijbs.40516)
Supplement: Supplementary file 1 — Supplementary figure and tables. [file ijbsv16p0739s1.pdf]

Supplementary Material

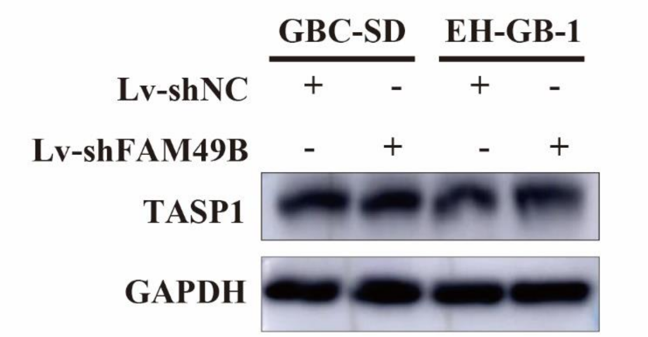

Figure S1. The expression correlation of TASP1 with FAM49B. The TASP1 expression level has no change when GBC-SD and EH-GB-1 cells were transfected with Lv-shNC and Lv-shFAM49B.

Tables

Table S1. Primers used for vectors construction

Table S2. Primers used for qRT-PCR

Table S1. Primers used for vectors construction

Name	Sequence
**pCMVPuro02-TASP1-F**	5'- CTAGTCTAGAGCCACCATGACCATGGAGAAGGGGATG -3'
**pCMVPuro02-TASP1-R**	5'- TCCTTCGAAGTTCACTGGGCTCTCCAGGC -3'
**GV141-FAM49B-F**	5'- ACGGGCCCTCTAGACTCGAGCGCCACCATGGGGAATCTTCTTAAAGTTTTG -3'
**GV141-FAM49B-R**	5'- AGTCCAGTGTGGTGGAATTCTTGCAGCATGGATTTAATTTGCTTG -3'

Table S2. Primers used for qRT-PCR

Name	Sequence
**TASP1-F**	5'- AGGCACTTTGGACACGGTAG -3'
**TASP1-R**	5'- CAAGCACGCCATCTTCACT -3'
**FAM49B-F**	5'- GCAGAAGGCATCTTGGAGGACTTG -3'
**FAM49B-R**	5'- ACCTCTTAATGCTGCTTCTAACCTCTG -3'
**GAPDH-F**	5'- AGAAGGCTGGGGCTCATTTG -3'
**GAPDH-R**	5'- AGGGGCCATCCACAGTCTTC -3'
